# Forkhead box O (FOXO) 3 modulates hypoxia-induced autophagy through AMPK signalling pathway in cardiomyocytes

**DOI:** 10.1042/BSR20160091

**Published:** 2016-06-17

**Authors:** Yunpeng Chi, Conghong Shi, Yang Zhao, Chengjun Guo

**Affiliations:** * Department of Cardiology, Beijing Anzhen Hospital, Capital Medical University, Beijing 100029, China; † Department of Cardiology, Baotou Fourth Hospital, Inner Mongolia Autonomous Region, Baotou 014030, China; ‡ Department of Cardiac Surgery, Beijing Anzhen Hospital, Capital Medical University, Beijing 100029, China

**Keywords:** AMP-activated protein kinase (AMPK), autophagy, cardiomyocytes, forkhead box O (FOXO) 3, hypoxia

## Abstract

Autophagy is promoted as a response to such environmental stress conditions as ATP depletion and excessive accumulation of reactive oxygen species (ROS). Multiple signalling pathways, including AMP-activated protein kinase (AMPK), are indicated to promote autophagy in ischaemic/hypoxic (I/R) heart. However, it's far more to clarify the orchestrated cross-talk between AMPK and other signalling pathways in the autophagy. In the present study, we investigated the autophagy induction by hypoxia in Rat H9C2 cardiomyocytes with LC3-EGFP reporter, EM and Western blot analysis. Then, we examined the promotion of forkhead box O (FOXO) 3, one member of FOXO transcriptional protein family, by hypoxia in Rat H9C2 cells and determined the mediation of FOXO 3 in the hypoxia-induced autophagy in H9C2 cells. In addition, we investigated the role of AMPK signalling in the FOXO3-mediated, hypoxia-induced autophagy in H9C2 cells. It was demonstrated that hypoxia induced significant autophagy in H9C2 cells, via promoting autophagic vesicles, inducing the conversion of LC3-I to LC3-II and up-regulating autophagy-related (ATG) markers. Moreover, FOXO3 was up-regulated by the hypoxia in H9C2 cells; and the knockdown of FOXO3 significantly reduced the hypoxia-induced autophagy. In addition, AMPK signalling was significantly promoted by hypoxia in H9C2 cells, and the chemical manipulation of AMPK exerted significant influence on the hypoxia-induced autophagy and on the FOXO3 level. In conclusion, FOXO3 regulated the hypoxia-induced autophagy in cardiomyocytes, and AMPK mediated the FOXO3 promotion during the autophagy induction by hypoxia, implying the key regulatory role of FOXO3 and AMPK signalling in the hypoxia-induced autophagy in cardiomyocytes.

## INTRODUCTION

Ischaemic/hypoxic (I/R) heart disease is widely conceived as the single leading cause of death worldwide. The percentage keeps in high of patients suffering from acute myocardial infarction (MI) and experiencing sudden cardiac death [1]. Up to now, we are far from clear understanding the mechanisms underlining the myocardial ischaemia/infarction, though there are accumulating and massive studies focusing on the subject. Apoptosis, as a form of programmed cell death, has been emphasized to mediate the cardiomyocyte death during ischaemic heart disease [2]. Autophagy is a dynamic self-degradation process for cellular components by cellular lysosome under a stringent regulation [3,4], and has also been recognized to involve in the ischaemic heart disease [5]. Autophagy normally maintains at a low level in heart, and is sharply promoted as a response to such environmental stress conditions as ATP depletion, excessive reactive oxygen species (ROS) and mitochondrial dysfunction [6,7].

Multiple signalling pathways have been indicated to mediate autophagy in the I/R heart. The reduced ATP has been recognized to activate autophagy [6]. The increased AMP/ATP ratio activates AMP-activated protein kinase (AMPK) [9] and successively induces autophagy via inhibiting mammalian target of rapamycin (mTOR) [6]. The promoted ROS in the myocardium of an ischaemic heart [10,11] can also activate autophagy in neonatal cardiomyocytes [12] via inhibiting the mitochondrial function [13]. However, it's hard to associate the multiple signals and to clarify the orchestrated cross-talk of them in the autophagy induction in cardiomyocyte under I/R condition.

Forkhead box O (FOXO) 3, one member of FOXO transcriptional protein family, has been confirmed to modulate autophagy [14,15]. FOXO 3 can activate autophagy via transcriptionally up-regulating autophagy-related (ATG) genes or autophagy regulatory genes, such as those genes encoding microtubule-associated protein 1 light chain 3 (MAP1LC3), autophagy-related 12 (ATG12), gamma-aminobutyric acid receptor-associated protein-like 1 (GABARAPL1) [homologue of yeast autophagy-related 8 (ATG8)] and BCL2/adenovirus E1B 19 kDa protein-interacting protein 3 (BNIP3) [14,15]. However, little is known about the upstream regulation of FOXO3 in autophagy. It was indicated that FOXO3 was required for starvation-induced autophagy [16]. And the FOXO3-mediated autophagy was also found in oxidative stress response to such pathological conditions as ischaemia/reperfusion and hypoxia in tumours [17]. Moreover, the activation of AMPK signalling has been confirmed to promote FOXO3 under oxidative stress [18,19].

In the present study, we investigated the autophagy induction by hypoxia in cardiomyocytes, and determined the regulatory role of FOXO3 in the hypoxia-induced autophagy. In addition, we examined the key modulation of AMPK signalling on the FOXO3 promotion by hypoxia. The present study brought insight into the implication of AMPK signalling in the FOXO3-mediated autophagy in cardiomyocytes, under hypoxia.

## MATERIALS AND METHODS

### Cell culture and treatment

Rat H9C2 cardiomyocytes were cultured in Dulbecco's Modified Eagle's Medium (DMEM), supplemented with 10% FBS (Invitrogen), 100 units/ml penicillin and 100 mg/ml streptomycin (CSPC Pharmaceutical Group) at 37°C. For hypoxia culture, cell flask or plate was placed in a hypoxia incubator infused with a gas mixture of 5% CO_2_, 2% oxygen and nitrogen and the normoxia culture was performed with a gas mixture of 5% CO_2_, 21% oxygen and nitrogen. To induce or inhibit the AMPK activity, 5-aminoimidazole-4-carboxamide-1-β-D-ribofuranoside (AICAR) and *N*-acetyl-L-cysteine (NAC) (Sigma–Aldrich) were added to the DMEM with 2% FBS to maintain H9C2 cells, with a concentration of 150 nM and 200 nM respectively.

### Quantitative GFP-LC3 analysis and EM

GFP-LC3 reporter [20] was used to quantify the autophagic vesicles formed in cardiomyocytes with various treatments. H9C2 cells with more than 85% confluence were transfected with the GFP-LC3 reporter plasmid with Lipofectamine 2000 (Invitrogen). Twenty-four hours later, cells were updated with DMEM medium supplemented with 2% FBS, with or without rapamycin (200 nM). For the hypoxia culture, cells were cultured in the hypoxia incubator for 8, 12 or 24 h. Then, the GFP-positive vesicles were visualized and counted under fluorescence microscopy. The autophagic vesicles in H9C2 cells were also visually examined by EM with a transmission electron microscope (JEM1230) [20].

### Western blotting assay

H9C2 cells, post-treatment, were harvested with a cell scratcher and were homogenized in an ice-cold Cell Lysis Buffer (CST). Cellular supernatant was collected post a centrifugation of 12000 × ***g*** for 30 min at 4°C. Each protein sample with equal amount was separated with 10% or 12% SDS/PAGE gel and was transferred to a PVDF membrane (Millipore). The membrane was successively blocked with 2% BSA (Ameresco) overnight at 4°C, incubated with the rabbit polyclone antibody [against LC3, hypoxia-inducible factor (HIF)-1α, mTOR, Atg7, FOXO3, AMP-activated protein kinase α (AMPKα) with or without phosphorylated Thr^172^, acetyl-CoA carboxylase (ACC) with or without phosphorylated Ser^79^ or β-actin] 4 h or overnight at 4°C, and incubated with horseradish peroxidase (HRP)-linked secondary anti-rabbit antibody for 1 h at room temperature. The specific binding band was scanned and quantified according to the band density by ImageJ software.

### FOXO3 knockdown via RNA interference

The FOXO3 siRNA oligonucleotides (25 nM) or the scrambler oligonucleotides as control (25 nM) were purchased from Thermo Fisher, and were transfected into H9C2 cells with Opti-MEM containing Lipofectamine RNAiMax (Invitrogen). Six hours post transfection, cells were updated with fresh DMEM medium, which was supplemented with 2% FBS, and were subject to other treatment or were assayed for the knockdown efficiency post another inoculation of 24 h.

### Intracellular ROS measurement

The ROS level was determined with the fluorescent probe dichlorofluorescein diacetate (DCFH-DA) (Sigma–Aldrich), which can be oxidized to the highly fluorescent compound 2′,7′-dichlorofluorescein (DCF). DCF-positive cells were observed and counted under a live cell imaging system (Olympus LCS SYSTEM) (excitation at 485 nm and emission at 530 nm).

### Statistical evaluations

Quantitative results are presented as mean ± S.E.M. For the analysis between two groups on the GFP-LC3 dots, the expression of each molecule, the DCFDA level, the Student's *t* test was performed. A *P* value less than 0.05 was considered significant.

## RESULTS

### Hypoxia induces autophagy in H9C2 cardiomyocytes

**Figure 1:**
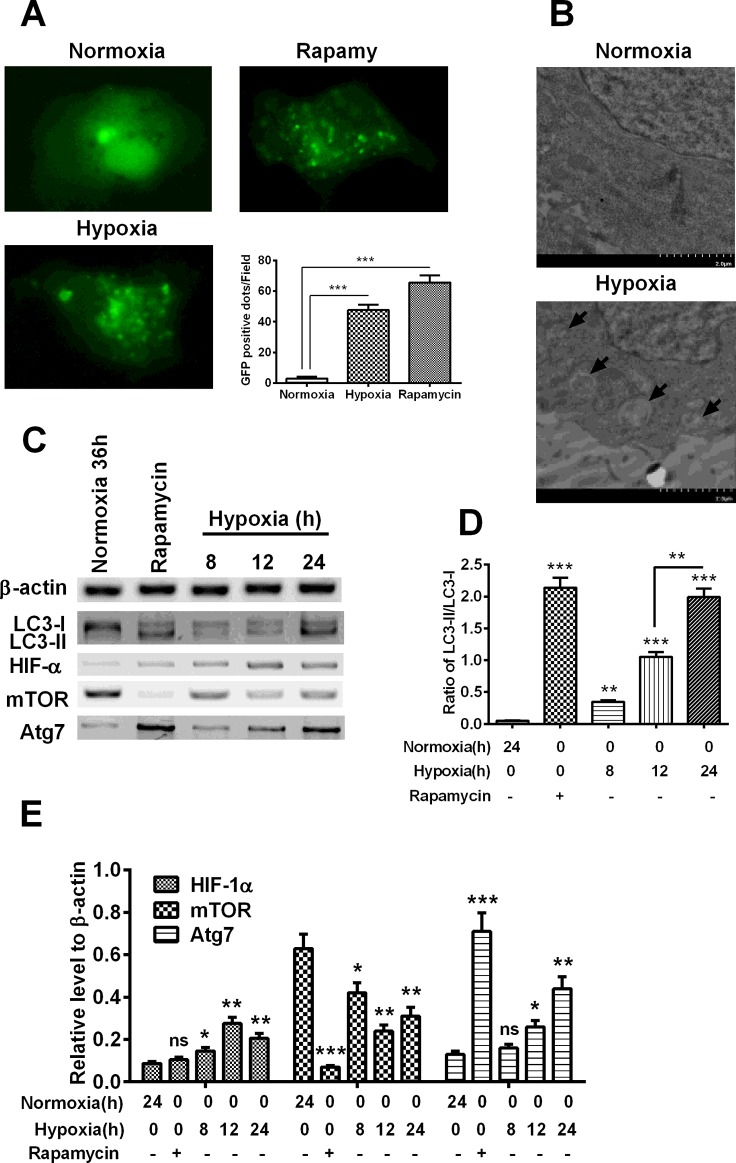
Hypoxia induces autophagy in cardiomyocytes (**A**) Representative images of GFP-LC3-positive vesicles in H9C2 cells subject to normoxia, hypoxia or the rapamycin treatment (200 nM), post the transfection with GFP-LC3 reporter. (**B**) Representative images of autophagic vesicles under EM in cardiomyocytes. (**C**) Western blot analysis of the conversion of LC3-I to LC3-II, the expression of HIF-1α, mTOR and Atg7 in the hypoxia-treated H9C2 cardiomyocytes. (**D**) Relative conversion level of LC3-I to LC3-II. (**E**) Percentage of HIF-1α, mTOR and Atg7 to β-actin. Experiments were repeated in triplicate; *P<0.05, **P<0.01, ***P<0.001; ns: no significance.

To determine the autophagy induction by hypoxia, we transfected GFP-LC3 reporter into H9C2 cardiomyocytes, and then incubated cells under hypoxia for 8, 12 or 24 h. As shown in Figure 1(A), there were significantly more GFP-LC3-positive autophagic vesicles, diffusely distributing in cytosol, in the H9C2 cells under hypoxia for 24 h, compared with the cells under normoxia (*P*<0.001). And such up-regulation of GFP-LC3-positive autophagic vesicles was also found in H9C2 cells which were treated with the autophagy inducer, rapamycin, with 200 nM (*P*<0.001). To confirm the autophagy induction by hypoxia, we then examined the autophagosome in the H9C2 cells under hypoxia via EM, the representative ultra-structures of the autophagosome under EM microphotography were found in H9C2 cells under hypoxia, rather than in H9C2 cells under normoxia (Figure 1B). Thus, we confirmed the induction of autophagy by hypoxia.

Then, we analysed the expression of ATG and autophagy-regulated genes, such as LC3, mTOR and Atg7 and the expression of HIF-1α, which is up-regulated by hypoxia [21]. It was indicated in Figures 1(C) and 1(D) that the conversion of LC3-I to LC3-II, which is the marker of autophagy [22], was significantly up-regulated by the rapamycin treatment (*P*<0.001) or the hypoxia treatment (*P*<0.01 for 8 h, *P*<0.001 for either 12 or 24 h), with a time-dependence (*P*<0.01 for the difference between 12 h and 24 h). However, the HIF-1α was only promoted by the hypoxia (*P*<0.05 or *P*<0.01), rather than normoxia or the treatment with rapamycin (Figures 1C and 1E). In addition, the mTOR was markedly reduced in H9C2 cells post the rapamycin treatment (*P*<0.001) or the incubation under hypoxia (*P*<0.05 or *P*<0.01). And the up-regulation of Atg7 was also confirmed in the rapamycin-treated (*P*<0.001) or hypoxia-treated H9C2 cells (*P*<0.05 for 12 h, *P*<0.01 for 24 h). Taken together, we confirmed the autophagy induction by hypoxia in H9C2 cardiomyocytes.

### FOXO3 mediates the hypoxia-induced autophagy in H9C2 cardiomyocytes

**Figure 2:**
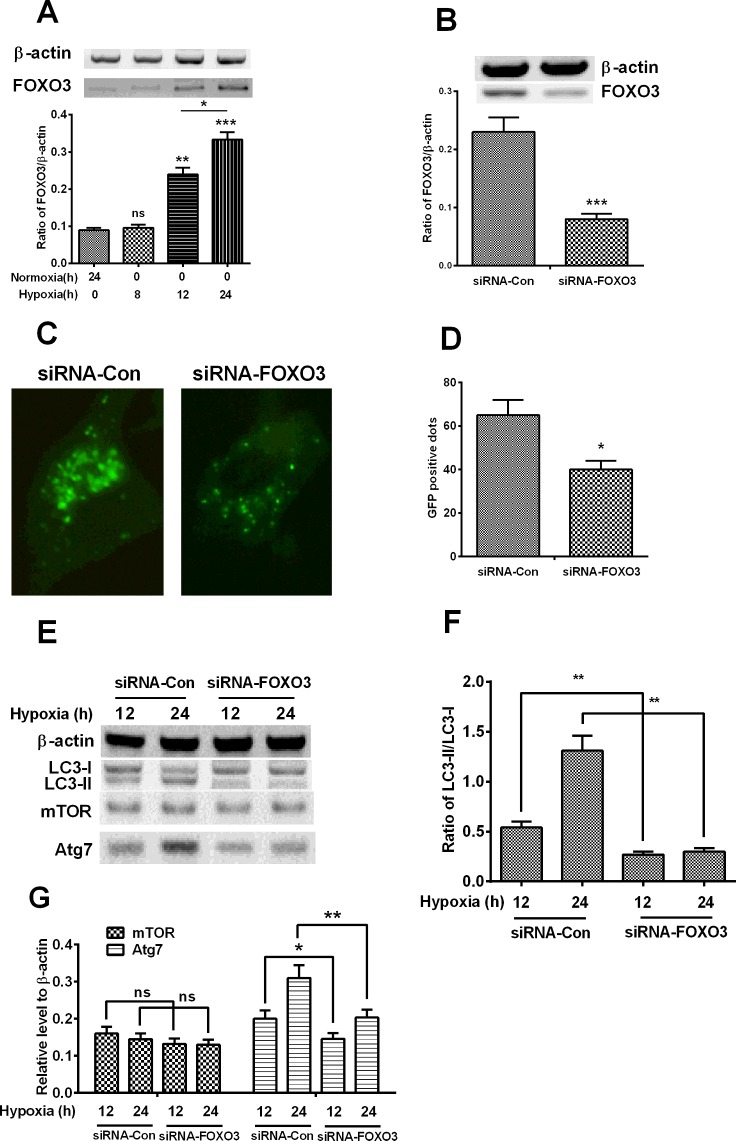
Hypoxia promotes FOXO3 which modulates hypoxia-induced autophagy (**A**) Western blot analysis of the significant promotion of FOXO3 in H9C2 cells subject to normoxia or hypoxia. (**B**) Expression of FOXO3 in hypoxia-treated H9C2 cells post the transient transfection with scramble RNA (siRNA-Con) or with siRNA–FOXO3. (**C** and **D**) Representative images (**C**) and quantified counting (**D**) of GFP-LC3-positive vesicles in hypoxia-treated H9C2 cells post transient transfection with scramble RNA (siRNA-Con) or with siRNA–FOXO3. (**E**) Western blot analysis of the conversion of LC3-I to LC3-II, the expression of mTOR and Atg7 in the hypoxia-treated H9C2 cells post the transient transfection with scramble RNA (siRNA-Con) or with siRNA–FOXO3. (**F** and **G**) Relative level of the conversion of LC3-I to LC3-II and the expression of mTOR and Atg7. The quantitative data were averaged for triple independent results; *P<0.05, **P<0.01, ***P<0.001; ns: no significance.

As a transcription factor, FOXO3 targets genes which are implicated in the atrophy [23] and in autophagy in cardiac myocytes [24], or in the inhibition of cell cycle progression in neonatal cardiomyocytes [25]. To further investigate whether FOXO3 is implicated in the hypoxia-induced autophagy in H9C2 cardiomyocytes, we investigated the induction of FOXO3 by hypoxia in H9C2 cells, and then examined the regulatory role of FOXO3 in the hypoxia-induced autophagy in H9C2 cells. It was shown in Figure 2(A) that the FOXO3 was significantly promoted by hypoxia for 12 or 24 h post-treatment (*P*<0.01 for 12 h and *P*<0.001 for 24 h), time-dependently (*P*<0.05 for the difference between the treatment for 12 h and 24 h). Then, we utilized the siRNA targeting FOXO3 to knockdown the hypoxia-promoted FOXO3. It was demonstrated that the promoted FOXO3 by hypoxia (for 24 h) could be markedly reduced by the transfection with 25 nM siRNA–FOXO3, compared with the control siRNA (siRNA-Con) (Figure 2B, *P*<0.001). Moreover, the hypoxia-induced autophagic vesicles were also significantly down-regulated by the siRNA–FOXO3 (Figures 2C and 2D, *P*<0.05).

The negative regulation of siRNA–FOXO3 on the hypoxia-induced autophagy was also examined by the Western blotting assay. Figures 2(E) and 2(F) indicated that the conversion of LC3-I to LC3-II was also inhibited by the siRNA–FOXO3 (*P*<0.01 for either 12 or 24 h post hypoxia). The induction of Atg7 by hypoxia was also negatively regulated by the siRNA–FOXO3, the expression of Atg7 was significantly lower in the siRNA–FOXO3 group than in the siRNA-Con group (Figure 2G, *P*<0.05 for 12 h, *P*<0.01 for 24 h post hypoxia). However, the down-regulation on the expression of mTOR was not significant between the two groups. Thus, these results confirmed that FOXO3 was up-regulated by hypoxia and involved in the hypoxia-induced autophagy in H9C2 cardiomyocytes.

### AMPK signalling was promoted by hypoxia in H9C2 cardiomyocytes

**Figure 3:**
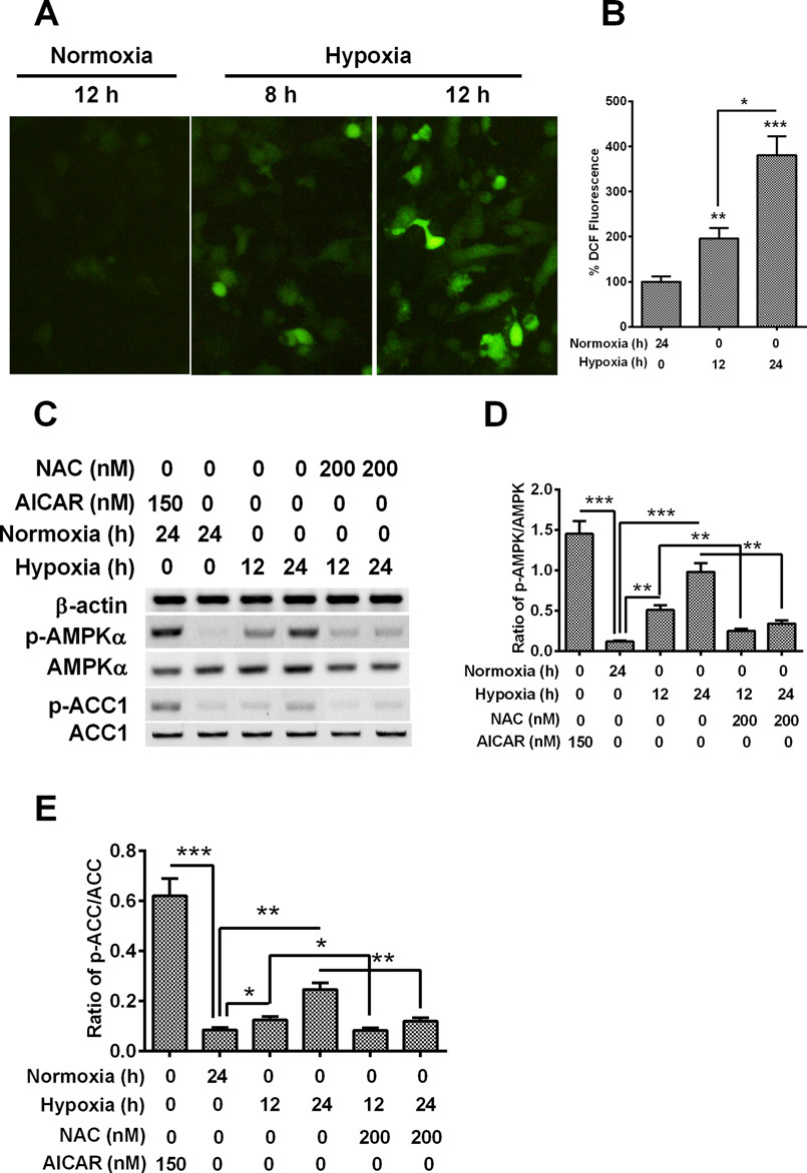
AMPK is activated by hypoxia via oxidant signalling (**A** and **B**) DCFDA detection for cellular ROS in H9C2 cells under normoxia or hypoxia, the representive image of DCF staining (**A**) and the quantitative analysis (**B**) was in indicated. (**C**) Western blotting of AMPK signalling in H9C2 cells under hypoxia and treated with AICAR or NAC. (**D** and **E**) Levels of AMPK phosphorylated at Thr^172^ (p-AMPK) (**D**) and of ACC phosphorylated at Ser^79^ (p-ACC) (**E**) respectively relative to AMPK and ACC, as was presented as fold change in the p-AMPK/AMPK or p-ACC/ACC ratio; *P<0.05, **P<0.01, ***P<0.001.

AMPK is a key regulator of energy homoeostasis [26], and has been previously suggested to regulate oxidative metabolism via regulating cellular energy homoeostasis through the autophagic recycling of intracellular components [6,27]. To further explore the signalling pathways in the hypoxia-induced autophagy in cardiomyocytes, we investigated the involvement of AMPK signalling in H9C2 cells under hypoxia. Firstly, we analysed the production of ROS in H9C2 cells under hypoxia with a DCFH-DA kit for ROS. It was shown in Figures 3(A) and 3(B) that the significantly promoted DCF fluorescence indicated a significant promotion of ROS by hypoxia in H9C2 cells (*P*<0.01for 8 h or *P*<0.001 for 12 h), time-dependently (*P*<0.05). Then, we analysed the expression and activation of two key regulators, AMPKα and ACC, in AMPK signalling cascades, by Western blotting assay. Figure 3(C) demonstrated that there was no marked promotion to AMPKα and ACC expression in hypoxia-treated H9C2 cells, whereas the activated forms of both regulators, AMPKα with a phosphorylated Thr^172^ and ACC with a phosphorylated Ser^89^ were significantly promoted by the hypoxia treatment in H9C2 cells. The ratio of p-AMPKα to AMPKα and the ratio of p-ACC to ACC were markedly higher in the hypoxia-treated cells than in the cells under normoxia (Figures 3D and 3E, *P*<0.01 or *P*<0.001 for ratio of p-AMPKα to AMPKα, *P*<0.05 or *P*<0.01 for ratio of p-ACC to ACC).

To reconfirm the activation of AMPK signalling by hypoxia, we treated H9C2 cells with chemical antioxidant NAC with a concentration of 200 nM before hypoxia. Interestingly, the promotion to p-AMPKα and p-ACC was significantly blocked by the NAC treatment. The ratio of p-AMPKα to AMPKα and the ratio of p-ACC to ACC were significantly reduced in the NAC-treated H9C2 cells than in non-treated cells (Figures 3C–3E, *P*<0.05 or *P*<0.01). In addition, to qualify the assay for AMPK signalling, we also treated H9C2 cells with a chemical AMPK activator, AICAR [28] and examined the activation of AMPK signalling in H9C2 cells. It was indicated that the level of AMPK activation was significantly promoted by the AICAR (Figures 3C–3E, *P*<0.001 for either p-AMPK or p-ACC). Therefore, the AMPK signalling was also induced by hypoxia in H9C2 cardiomyocytes.

### AMPK mediates the FOXO3 up-regulation and autophagy by hypoxia

**Figure 4:**
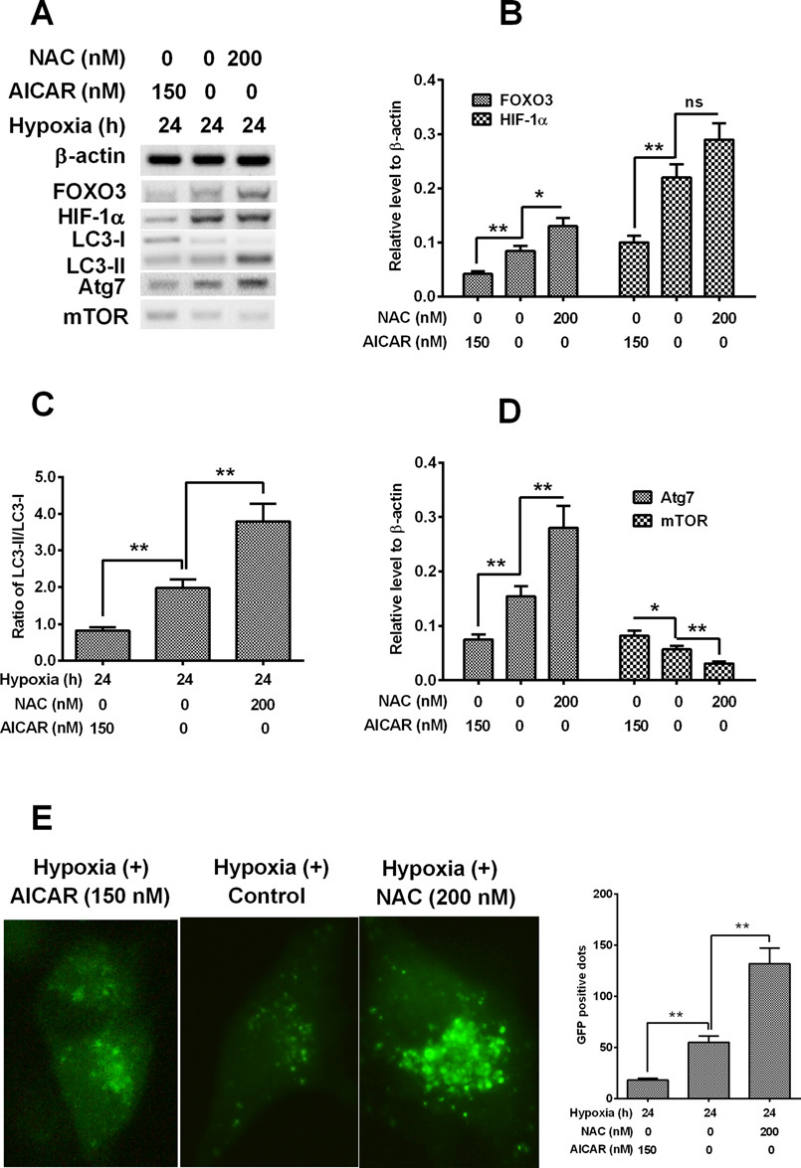
AMPK mediates the FOXO3 up-regulation by hypoxia (**A**) Western blot analysis of ATG or autophagy-regulated markers in H9C2 cells post the treatment with AMPK inhibitor, NAC or with AMPK activator, AICAR, under hypoxia. (**B**) Relative level of FOXO3 and HIF-1α in the NAC- (200 nM) or AICAR- (150 nM) treated H9C2 cells under hypoxia. (**C**) Conversion of LC3-I to LC3-II in the above-mentioned H9C2 cells. (**D**) Relative level of Atg7 and mTOR in the above-mentioned H9C2 cells. (**E**) Representative images of GFP-LC3-positive vesicles in H9C2 cells subject to above-mentioned treatment, post the transfection with GFP-LC3 reporter, the GFP-LC3-positive autophagic vesicles were indicated; *P<0.05, **P<0.01; ns: no significance.

To further identify the role of AMPK in the FOXO3 up-regulation and in the autophagy induction in H9C2 cells, we then investigated the influence of AMPK promotion or inhibition on the expression FOXO3, ATG or regulatory molecules in H9C2 cells subject to hypoxia. It was indicated in Figures 4(A) and 4(B), that the chemical inhibition of AMPK by NAC significantly inhibited the hypoxia-induced FOXO3 in H9C2 cells, whereas the AICAR treatment accelerated such induction of FOXO3. And the HIF-1α was also negatively or positively regulated by the treatment with NAC or with AICAR. Moreover, as shown in Figure 4(C), the hypoxia-promoted conversion of LC3-I to LC3-II was also regulated by the manipulation of AMPK activity with NAC or AICAR, with a blocked conversion in NAC-treated cells, whereas with an accelerated conversion in the AICAR-treated cells. In addition, the influence by AMPK manipulation was also confirmed in the induction of Atg7 and mTOR (Figure 4D).

And we reconfirmed the influence by NAC or by AICAR on the hypoxia-induced autophagy with the assay with GFP-LC3 reporter. It was indicated in Figure 4(E), there were significantly reduced GFP-LC3-positive autophagic vesicles in the NAC-treated H9C2 cells, whereas there were markedly aggravated promotion of such vesicles in the AICAR-treated H9C2 cells (*P*<0.01 respectively).

## DISCUSSION

I/R heart disease include a series of events, such as microvascular damage and cardiomyocyte death [29,30]. Autophagy is indicated to be promoted as a response to the ATP depletion and to the ROS accumulation in I/R heart disease [6,7]. The present study confirmed the autophagy induction by hypoxia in cardiomyocytes via various methods, and found a significant promotion of ATG markers such as LC3-II and Atg7, whereas the mTOR was significantly reduced in the hypoxia-treated cardiomyocytes. In addition, the HIF-1α was also up-regulated by the hypoxia. FOXO3 can activate autophagy by transcriptional up-regulation of ATGs or autophagy regulatory genes [23,31] in cardiomyocytes. And in the present study, we found a significant promotion of FOXO3 by hypoxia along with the autophagy induction. Moreover, the FOXO3 involved in the hypoxia-induced autophagy, FOXO3 knockdown via siRNA significantly reduced the formation of autophagic vesicles, down-regulated the conversion of LC3-I to LC3-II and reduced the expression of Atg7. Interestingly, the mTOR was not significantly regulated by the FOXO3 knockdown.

Up to now, it is not clear about the mechanism underlining the regulation in cardiomyocytes on the FOXO3-mediated autophagy, which was found in oxidative stress response to ischaemia/hypoxia [17]. Our study then investigated the activation of AMPK signalling in response to hypoxia in cardiomyocytes, and then evaluated the regulatory role of AMPK in the FOXO3 promotion and in the autophagy induction by hypoxia. Our results confirmed the promotion of AMPK signalling in H9C2 cells, under hypoxia, with significant up-regulation of AMPK phosphorylated at Thr^172^ (p-AMPK) and of ACC phosphorylated at Ser^79^ (p-ACC). Moreover, activation of AMPK signalling has been confirmed to promote FOXO3 under oxidative stress [18,19]. In addition, the hypoxia-induced FOXO3 was markedly reduced by the AMPK inhibitor, NAC, whereas was up-regulated by the AMPK activator, AICAR, implying the regulatory role of AMPK signalling in the FOXO3-mediated, hypoxia-promoted autophagy in cardiomyocytes. And, the negative regulation by NAC and the positive regulation by AICAR were also found in the promotion to such ATG or autophagy-regulated markers as LC3-II, mTOR, Atg7. Thus, the present study confirmed the regulatory role of AMPK in the FOXO3-mediated, hypoxia-promoted autophagy in cardiomyocytes.

In addition, the regulatory roles of several other signalling markers needed to be identified in the hypoxia-induced apoptosis. Our results indicated that the mTOR was down-regulated by hypoxia in H9C2 cells, and the manipulation of AMPK activity also exerted regulation on mTOR in H9C2 cells under hypoxia. However, the knockdown of FOXO3 exerted no influence on the mTOR level, implying no regulation of FOXO3 on the mTOR signalling in the hypoxia-induced autophagy. And given the wide range of ATG and autophagy-regulated markers were regulated by the AMPK signalling in the hypoxia-induced autophagy in cardiomyocytes, it seems that the FOXO3 signalling was only one branch pathway in the mediation of AMPK signalling in the hypoxia-induced autophagy in cardiomyocytes.

In conclusion, the present study confirmed the regulatory role of FOXO3 in the hypoxia-induced autophagy in cardiomyocytes. Moreover, our study indicated that AMPK regulated the FOXO3 promotion during the autophagy induction by hypoxia. In addition, our study indicated the wide range of regulation of AMPK in the hypoxia-induced autophagy in cardiomyocytes. However, further studies are necessary to explore more signalling pathways that link the regulation of autophagy and the AMPK signalling.

## Abbreviations

ACC: acetyl-CoA carboxylase
AICAR: 5-aminoimidazole-4-carboxamide-1-β-D-ribofuranoside
AMPK: AMP-activated protein kinase
AMPKα: AMP-activated protein kinase α
ATG: autophagy-related
BNIP3: BCL2/adenovirus E1B 19 kDa protein-interacting protein 3
DCF: 2',7'-dichlorofluorescin
DCFH-DA (DCFDA): dichlorofluorescein diacetate
DMEM: Dulbecco's Modified Eagle's Medium
FOXO: forkhead box O
GABARAPL1: gamma-aminobutyric acid receptor-associated protein-like 1
HIF: hypoxia-inducible factor
HRP: horseradish peroxidase
I/R: ischaemic/hypoxic
MAP1LC3 (LC3): microtubule associated protein 1 light chain 3
MEM: Eagle's Medium
mTOR: mammalian target of rapamycin
NAC: *N*-acetyl-L-cysteine
ROS: reactive oxygen species
siRNA-Con: control siRNA
